# Large-Volume Liposuction and Manual Extraction in Lipedema: A Hybrid approach

**DOI:** 10.1007/s00266-025-05329-2

**Published:** 2025-10-27

**Authors:** Giw Mostofizadeh-Haghighi, Adrian Zaharie, Mojtaba Ghods

**Affiliations:** 1 Department of Plastic, Aesthetic and Reconstructive Microsurgery / Handsurgery, Hospital Ernst von Bergmann, Charlottenstraße 72, 14467 Potsdam, Germany; 2 Department of Plastic, Aesthetic and Reconstructive Microsurgery / Handsurgery, Hospital Ernst von Bergmann, Bad Belzig, Germany; 3 Center of Sports Medicine, University of Potsdam, Potsdam, Germany

**Keywords:** Lipedema, Liposuction, Lipoedema, Megaliposuction, Manual extraction

## Abstract

**Introduction:**

Lipedema is a chronic and potentially progressive fat distribution disorder. Disease-related symptoms, such as pain and discomfort, can require surgical intervention when conservative therapies are exhausted. These megaliposuctions are functional in nature and need to be distinguished from esthetic liposuctions. This new surgical approach, the hybrid technique combining power-assisted liposuction (PAL) with manual extraction (ME), has been developed to more effectively treat fibrotic nodules, particularly in the lower legs, where conventional liposuction techniques often fall short.

**Methods:**

A total of 24 patients with advanced lipedema were included in a matched cohort analysis: 12 treated with the hybrid technique and 12 treated with PAL alone. Patients were matched by age (*p*= 0.9416) and BMI (*p*=0.6489). Outcome measures included pain and heaviness reduction on the visual analog scale (VAS), changes in a comprehensive lipedema symptom score, intraoperative blood loss, complication rates, and total lipoaspirate volume.

**Results:**

While statistical significance was not reached, the hybrid group demonstrated a trend to greater symptom relief, with median reductions in pain (6.5 vs. 5.0, *p*= 0.1958), leg heaviness (7.5 vs. 6.5; *p*= 0.2293), and composite symptom score (68.0 vs. 52.5, *p*= 0.3254) compared to the PAL group. The hybrid technique also resulted in fewer postoperative complications and enabled the removal of larger total aspirate volumes; Hybrid group (mean: 17.88 liters), PAL group (mean: 16.62 liters) without increased risk.

**Conclusion:**

The hybrid approach shows promising trends in clinical outcomes and safety compared to PAL alone. Despite the absence of statistical significance due to the limited sample size, the consistent directional improvements and reduced complication rates support the further evaluation of this method in larger, prospective studies.

**Level of Evidence V:**

This journal requires that authors assign a level of evidence to each article. For a full description of these Evidence-Based Medicine ratings, please refer to the Table of Contents or the online Instructions to Authors www.springer.com/00266.

**Supplementary Information:**

The online version contains supplementary material available at 10.1007/s00266-025-05329-2.

## Introduction

Lipedema is a chronic and potentially progressive disorder of subcutaneous fat distribution that primarily affects women, typically manifesting during or after puberty [1]. It is characterized by the symmetrical accumulation of painful adipose tissue in the extremities, sparing the hands and feet. The disease is subdivided into clinical stages, with Stage III marked by large nodular changes and extensive fibrotic tissue, particularly in the lower legs [2]. While conservative treatment including compression therapy and manual lymphatic drainage remains the first line of management, many patients ultimately require surgical intervention to achieve sufficient symptom relief [3].

Power-assisted liposuction (PAL) is widely regarded as the gold standard surgical treatment for lipedema [4], providing substantial volume reduction and symptom improvement [5]. However, in advanced disease, particularly in the lower legs where fibrotic nodules [6] often lie directly beneath the dermis, standard liposuction techniques may prove insufficient. Excessive superficial suctioning in these areas risks both hemorrhage and postoperative skin irregularities.

To address these limitations, we propose a hybrid technique that combines large volume PAL with adjuvant manual extraction (ME). This method allows for the targeted removal of subdermal fibrotic nodules that resist aspiration and may otherwise compromise both the functional and esthetic outcomes of surgery. In this study, we outline our surgical technique and present comparative outcomes from a matched cohort of patients treated with the hybrid technique versus PAL alone.

## Method

**Fig. 1:**
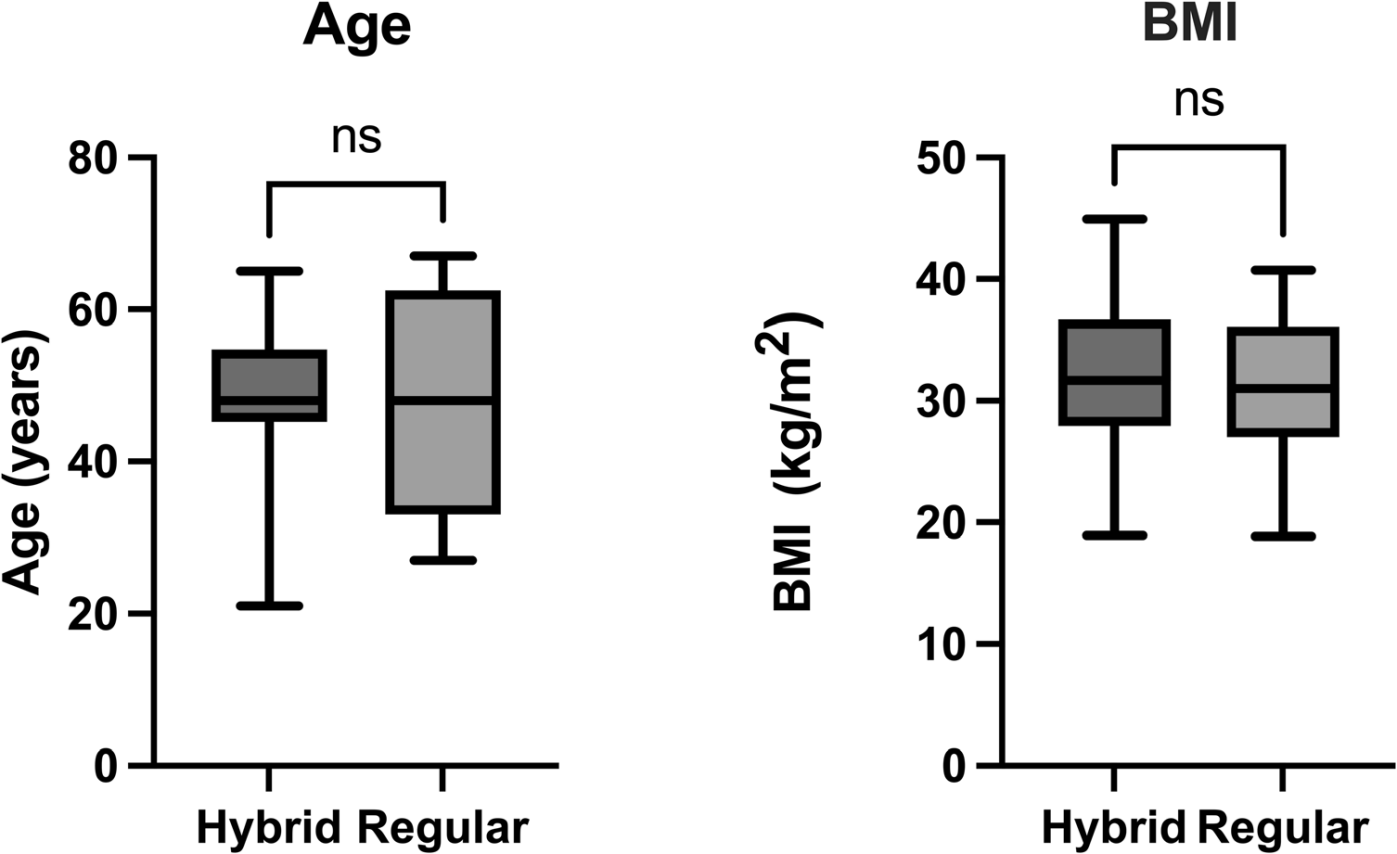
Comparison of baseline characteristics between treatment groups. Box plots showing patient age (left) and body mass index (BMI, right) for the hybrid liposuction and power-assisted liposuction (PAL) groups (n = 12 each). The boxes indicate the interquartile range (IQR, 25th–75th percentile), the horizontal line represents the median, and whiskers extend to the minimum and maximum values. No significant differences were observed between groups (paired t-test)

Patients were included if they had a confirmed clinical diagnosis of Stage II or III lipedema based on standardized criteria and if conservative therapy had been exhausted. Conservative therapy consisted of at least six months of complex physical decongestive therapy (CPDT), including daily use of flat knit class II compression garments and manual lymphatic drainage two to three times per week. Additional inclusion criteria were age between 21 and 67 years and a BMI between 18.5 and 45 kg/m^2^ (Fig. 1). Patients were offered the hybrid approach when prominent nodular structures were present in the pretibial region or on the dorsal aspect of the lower leg. Controls were selected using matched-pair methodology to ensure comparable baseline characteristics in terms of BMI and age. Patients with significant comorbidities such as uncontrolled diabetes, severe varicosis, or coagulopathies were excluded unless these conditions had been adequately treated prior to surgery. Hemoglobin and coagulation levels were evaluated preoperatively to assess perioperative risk.

Twelve patients underwent hybrid liposuction, while twelve age- and BMI-matched controls underwent conventional PAL. Each patient underwent two staged liposuction procedures for either anterior or posterior leg compartments. Standardized markings and photographic documentation were used preoperatively. In both groups, tumescent infiltration was performed using isotonic saline with epinephrine, followed by a 30-minute exposure time.

**Fig. 2:**
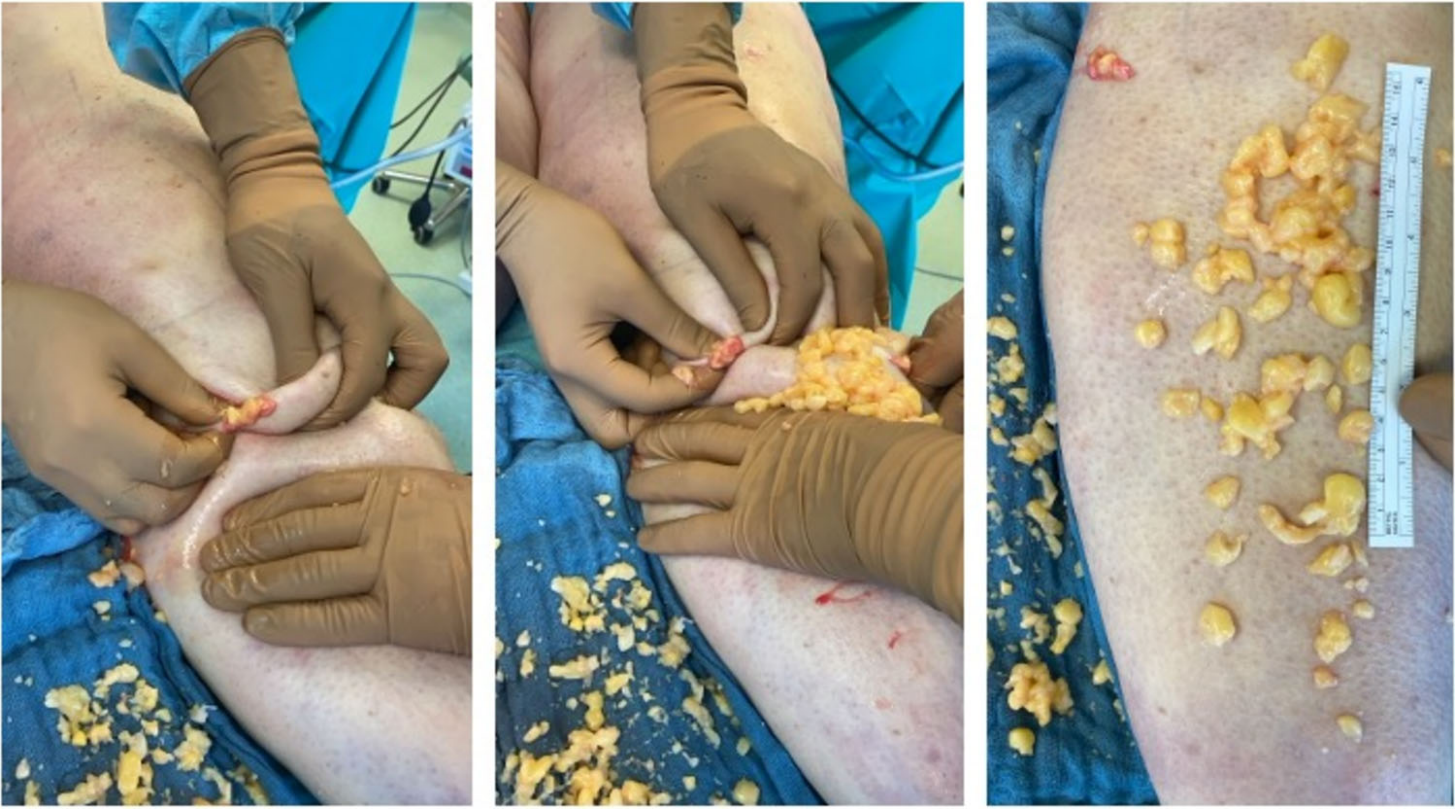
Intraoperative photographs demonstrating the manual extraction of fibrotic lipedema fat from the lower leg. After tissue emulsification and incision extension, resistant subcutaneous nodules are gently expressed using manual pressure. The extracted nodules typically exhibit a firm, lobulated morphology characteristic of advanced-stage lipedema. A metric scale (right panel) illustrates the size and quantity of evacuated tissue

In the hybrid group, deep layer debulking was first performed with a 5-mm Tri-Port PAL cannula, followed by superficial refinement with a 4-mm Mercedes cannula. Liposuction is performed in an axial direction to prevent damage to the lymphatic system [7]. Once the aspirate showed increased blood content or failed to yield further adipose tissue despite adequate pinch test (≥ 2 cm), the procedure was transitioned to manual extraction. Manual extraction was facilitated by emulsifying tissue with PAL motion (without suction) and extending the incision by 1 cm. Loose subcutaneous tissue was manually evacuated and modulated to smooth contours and access nodules otherwise inaccessible to suction (Fig. 2). A 3 mm cannula was then used for light superficial liposuction to stimulate skin retraction [8, 9].

The decision to convert to manual extraction was supported by visual assessment of the aspirate, where aspirate hematocrit was estimated at >10% or macroscopically dominated by blood rather than fat. Endoscopic inspection of the subcutaneous tissue was occasionally performed and revealed preserved vascular and lymphatic structures following hybrid treatment.

Compression garments (flat knit class II) were applied intraoperatively, followed by short stretch bandages on the lower legs. Manual lymphatic drainage was initiated on postoperative day one and continued three times per week. Patients were mobilized as early as possible and received standard postoperative instructions for wound care and pain management. Patient-reported outcome measures were collected at least 6 months postoperatively using scales for pain, heaviness, and a non-validated comprehensive lipedema symptom score (Table 1). We decided to change the scale of the last question. In the original questionnaire, the question was: “How satisfied are you with the appearance of your legs?”. We asked the question the other way around: “How unsatisfied are you with the appearance of your legs?” to obtain graphs that go in the same direction [10]. The distribution of the collected data was assessed and demonstrated a non-normal pattern; therefore, nonparametric statistical tests (Mann–Whitney U test) were applied for group comparisons.

**Table 1:** Questionnaire items used to assess the composite symptom score for lipedema

Question	Before the first surgery	Currently
How painful were the legs?	____	____
How was tenderness in the legs?	____	____
Were the legs prone to bruising?	____	____
Was there a feeling of tension in the legs?	____	____
Was there a feeling of warmth in the legs?	____	____
Was there a feeling of coldness in the legs?	____	____
Did you suffer from muscle cramps?	____	____
Did you have the feeling of “heavy legs”?	____	____
Did you have the feeling of “tired legs”?	____	____
Was there any swelling in the legs?	____	____
Were there any skin complications in the legs?	____	____
Did you suffer from itching?	____	____
Were there any limitations while walking?	____	____
How do you assess the impairment lipedema had on your quality of life?	____	____
How unsatisfied were you with the appearance of the legs?	____	____

Patients rated 15 symptom domains before the first surgery and at the time of follow-up, covering pain, tenderness, bruising, sensory changes, swelling, mobility, skin health, and quality of life. Responses formed the basis for calculating total symptom burden and treatment-related improvements

## Results

**Fig. 3:**
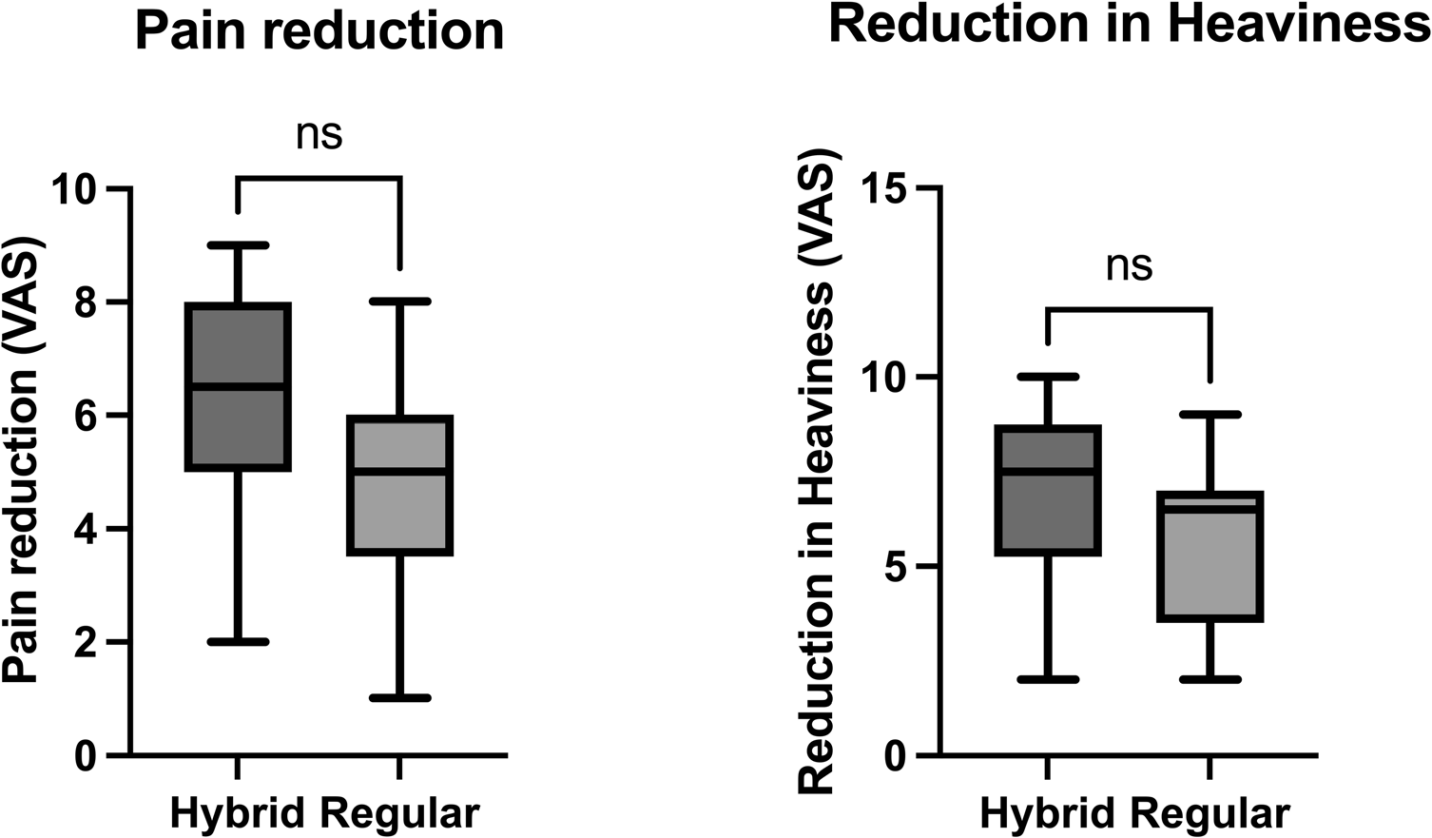
Patient-reported outcomes after surgical treatment. Box plots illustrating changes in pain (left) and leg heaviness (right) at 12-month follow-up, comparing the hybrid liposuction and PAL groups (n = 12 each). Each box shows the IQR, with the median represented by a horizontal line and whiskers indicating the full range. Although the hybrid group showed numerically greater symptom reductions, differences were not statistically significant (Mann–Whitney U test)

**Fig. 4:**
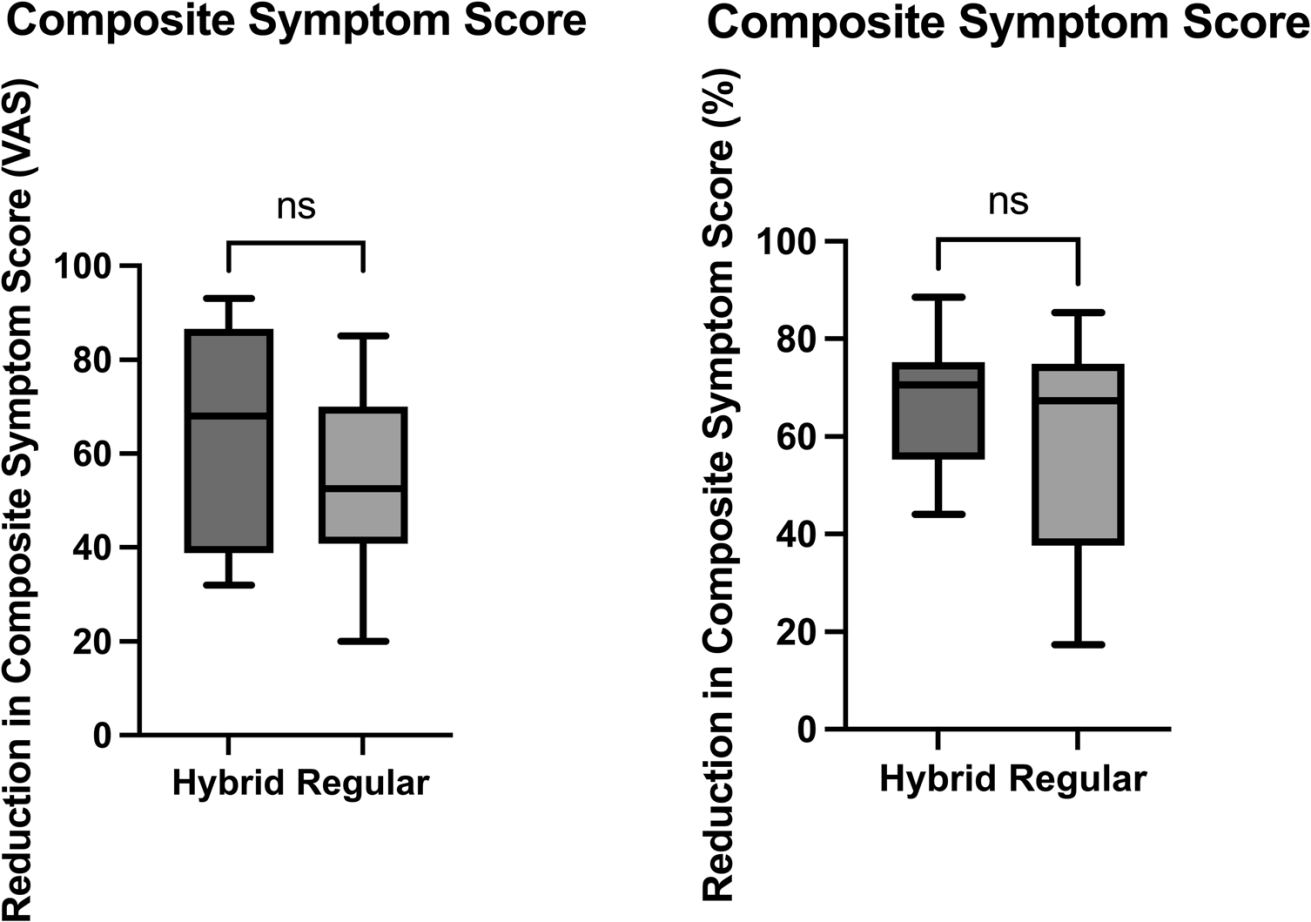
Reduction in composite symptom scores following liposuction. Box plots depicting absolute (left) and percentage (right) reductions in total lipedema symptom burden for patients treated with hybrid liposuction versus PAL (n = 12 each). Data are represented as medians with interquartile ranges and whiskers from minimum to maximum values

The hybrid group demonstrated greater median reductions in pain (6.5 vs. 5.0 points; *p* = 0.1958) (Fig. 3), leg heaviness (7.5 vs. 6.5 points; *p* = 0.2293) (Fig. 3), and composite symptom score (68.0 vs. 52.5 points; *p* = 0.3254) (Fig. 4) compared to the PAL group.

Intraoperative blood loss was comparable between groups (median 2.35 mmol per liter in Hybrid vs. 2.50 mmol per liter in PAL; *p* = 0.2710), with no clinically relevant difference observed. Among the 24 patients undergoing 48 surgeries, one hybrid patient required a prolonged hospital stay, whereas four PAL patients experienced extended hospitalizations. Total lipoaspirate volume was slightly higher in the hybrid group (mean 17.88 liters) than in the PAL group (mean 16.62 liters), suggesting that more complete debulking can be achieved without added risk.

## Conclusion

The hybrid technique combining power-assisted liposuction with manual extraction demonstrates favorable trends in symptom reduction, tissue debulking, and complication profile when compared to PAL alone. Patients treated with the hybrid method reported greater improvements in pain, leg heaviness, and composite symptom scores, alongside fewer postoperative complications and higher aspirate volumes. Although statistical significance was not achieved due to the limited sample size, the consistent clinical trends indicate that this technique may offer meaningful advantages for selected patients, particularly in advanced stages of lipedema with pronounced fibrosis. These findings support the continued use and further investigation of the hybrid method in larger, prospective studies aimed at validating its efficacy and long-term outcomes.

## Discussion

The hybrid technique, combining power-assisted liposuction with manual extraction, offers a promising alternative for the treatment of advanced lipedema, particularly in cases with dense fibrotic tissue that is poorly responsive to conventional aspiration alone. This study demonstrates consistent numerical advantages for the hybrid approach across multiple clinical domains, including reductions in pain, leg heaviness, and composite symptom score. Although statistical significance was not reached, likely due to the limited sample size, the observed trends suggest that the hybrid method can achieve more comprehensive tissue debulking and symptom relief without increasing the risk of complications. The consistent trends across clinical outcomes and complication profiles support the potential benefit of the hybrid method and justify further investigation.

One of the key benefits of the hybrid technique is its ability to address nodular and fibrotic fat deposits in the lower leg, which are often inaccessible to standard liposuction cannulas. By combining deep liposuction with manual evacuation of resistant tissue, the procedure enables more complete volume reduction while minimizing trauma to subdermal vascular and lymphatic structures. The preservation of these structures was confirmed in some cases through intraoperative endoscopic evaluation, which showed intact vessels and lymphatics post-extraction.

Complication rates were also favorable in the hybrid cohort. Only one hybrid patient required extended hospitalization, compared to four prolonged stays in the PAL group. This supports the notion that, when carefully performed, the hybrid approach does not compromise safety and may even reduce the risk of postoperative morbidity.

Despite these strengths, several limitations must be acknowledged. This study was limited by its small sample size, which restricts the ability to detect statistically significant differences despite clinically meaningful trends. Additionally, esthetic outcomes and symptom relief were assessed through patient-reported measures, which, although valuable, are inherently subjective. The procedure is also more labor intensive, requiring greater surgical expertise and time, which could limit its scalability in high volume centers.

Compared to other modalities such as water assisted liposuction (WAL) or VASER, the hybrid approach has not yet been directly benchmarked in large trials. However, the ability to mechanically disrupt and evacuate fibrotic tissue under direct control may offer advantages over purely device-based solutions. The integration of manual techniques into lipedema surgery warrants further exploration and standardization.

Ultimately, the findings support the clinical utility of the hybrid technique for selected patients with advanced lipedema. Larger, prospective studies are needed to confirm these outcomes, establish reproducibility across centers, and optimize patient selection criteria. With further validation, the hybrid method may become a standard component of the surgical armamentarium for managing fibrotic lipedema.

## Supplementary Information

Below is the link to the electronic supplementary material.

Supplementary file1 (MOV 151004 KB)
